# The crosstalk between mitochondrial dysfunction and fatty acid metabolism in heart failure: mechanisms and therapeutic strategies

**DOI:** 10.3389/fphar.2025.1679085

**Published:** 2025-10-09

**Authors:** Min Wang, Zheqin Zhu, Xuan He, Sisi Dai, Rongzhen Liu, Jianhe Liu

**Affiliations:** ^1^ The First Hospital of Hunan University of Chinese Medicine, Changsha, China; ^2^ College of Traditional Chinese Medicine, Changsha Medical University, Changsha, China; ^3^ Hunan University of Traditional Chinese Medicine, Changsha, China

**Keywords:** heart failure, mitochondrial dysfunction, fatty acid oxidation, energy metabolism, traditional Chinese medicine

## Abstract

Heart failure is characterized by progressive energetic insufficiency, in which mitochondrial dysfunction and impaired fatty acid oxidation are central features. Normally, the FAO provides most of the cardiac ATP supply, but in HF, this pathway becomes disrupted, leading to the accumulation of lipid intermediates, oxidative stress, and reduced ATP production. Emerging evidence suggests that mitochondrial impairment and FAO disturbances may interact reciprocally, forming a vicious cycle that aggravates energetic failure and structural remodeling. This review summarizes current knowledge on the bidirectional relationship between mitochondrial dysfunction and FAO abnormalities in HF. We integrate findings from experimental models with clinical observations that highlight the translational relevance of this interplay. In addition, we provide an updated overview of therapeutic strategies, including pharmacological modulators such as SGLT2 inhibitors and trimetazidine, as well as traditional Chinese medicine formulas such as Qiliqiangxin and Qishen granules, which have shown preliminary benefits in clinical studies. Although the proposed vicious cycle remains a working hypothesis requiring further validation, understanding this interplay may help identify novel biomarkers, stratify patients by metabolic phenotype, and guide precision therapies for HF.

## Graphical Abstract

**Figure:**
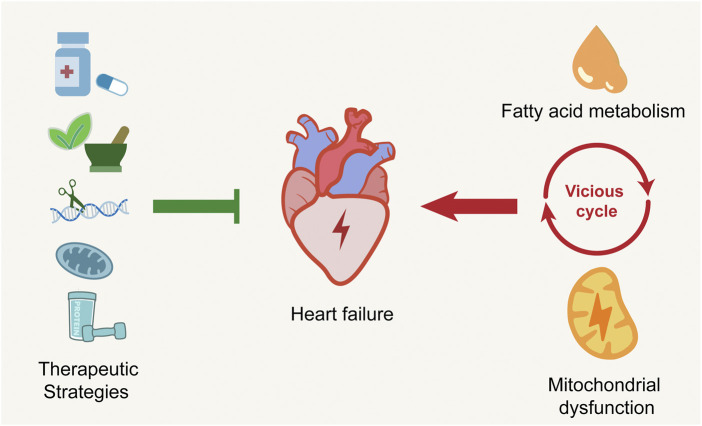

## 1 Introduction

Heart failure (HF) is the final stage of various cardiovascular diseases, affecting more than 26 million individuals worldwide, with a 5-year mortality rate nearing 50% (Khan et al., 2024). Alongside symptoms such as shortness of breath, fatigue, and peripheral swelling, which significantly diminish patients’ quality of life, HF also places a substantial economic burden on families and healthcare systems due to its high rates of recurrence and rehospitalization (McDonagh et al., 2023; McDonagh et al., 2024). Current clinical treatment mainly relies on pharmacological therapy, including β-adrenergic receptor blockers, renin-angiotensin-aldosterone system (RAAS) inhibitors, and sodium-glucose cotransporter 2 (SGLT2) inhibitors (Beghini et al., 2025). Many patients continue to experience recurrent hospitalizations, poor quality of life, and progressive decline in cardiac function (Kemp and Conte, 2012). These clinical realities underscore the urgent need for new mechanistic insights and therapeutic strategies beyond conventional neurohumoral modulation.

As a highly energy-demanding organ, the heart relies heavily on mitochondria to continually generate adenosine triphosphate (ATP) through oxidative phosphorylation (OXPHOS), with fatty acid oxidation (FAO) supplying about 60%–90% of this energy (Da Dalt et al., 2023). During the progression of HF, a major shift in myocardial metabolism occurs, marked by impaired fatty acid utilization, increased glycolysis, reduced mitochondrial biogenesis, and decreased OXPHOS efficiency (Yamamoto and Sano, 2022). Clinical studies employing positron emission tomography and magnetic resonance spectroscopy have confirmed that metabolic derangements correlate with disease severity and prognosis, highlighting myocardial “energy starvation” as a central hallmark of HF.

Mitochondrial dysfunction and disordered FAO are not isolated phenomena but appear to interact in a harmful cycle. On the one hand, defective mitochondrial electron transport impairs FAO flux, leading to the accumulation of toxic lipid intermediates and excessive reactive oxygen species (ROS) (Guo et al., 2018). On the other hand, dysregulated FAO further exacerbates mitochondrial injury through lipotoxicity, oxidative stress, and membrane destabilization (Sun et al., 2024). Together, these processes compromise ATP generation, aggravate structural remodeling, and accelerate HF progression (Wang et al., 2021). Importantly, clinical observations have shown that elevated plasma free fatty acids, increased lipid intermediates, and markers of oxidative stress are associated with adverse outcomes in HF patients, emphasizing the translational significance of this interplay.

Several reviews have recently addressed either mitochondrial dysfunction or metabolic abnormalities in HF. For example, Da Dalt et al. (2023) and Yamamoto and Sano (2022) focused primarily on fatty acid metabolism, whereas Gallo et al. (2024) and Zhou and Tian (2018) emphasized mitochondrial dysfunction as a pathophysiological driver. While these works have advanced our understanding, few reviews have systematically explored the bidirectional crosstalk between mitochondrial impairment and FAO dysregulation. Moreover, little attention has been paid to integrating conventional pharmacological therapies with traditional Chinese medicine (TCM) and natural products, despite their growing recognition in the management of cardiovascular diseases.

To address these gaps, the present review provides a comprehensive synthesis of current knowledge on the reciprocal relationship between mitochondrial dysfunction and FAO disturbances in HF. We summarize key molecular mechanisms underlying this vicious cycle, evaluate their translational relevance, and highlight potential therapeutic targets. In addition, we present updated evidence on both pharmacological agents and TCM-based interventions that act on mitochondrial energy metabolism. By integrating mechanistic insights with therapeutic perspectives, this article aims to contribute to the development of precision-targeted strategies for HF management.

## 2 Metabolic characteristics and mitochondrial function in the normal heart

### 2.1 Central role of mitochondria in myocardial energy metabolism

Mitochondria serve a dual role as the “powerhouse” and “signaling hub” of cardiomyocytes, occupying a central and irreplaceable position in myocardial energy metabolism. The heart’s continuous and rhythmic cycles of contraction and relaxation require a stable and substantial energy supply, primarily provided by mitochondria through OXPHOS (Stoldt et al., 2018). OXPHOS is a highly complex and tightly regulated physiological process that occurs on the inner mitochondrial membrane (Benard et al., 2006). This membrane hosts a series of respiratory chain complexes, each made up of multiple protein subunits.

At the core of mitochondrial function lies the electron transport chain (ETC.), composed of complexes I–IV and ATP synthase (complex V) (Green, 1983; Sharma et al., 2009; Wu et al., 2016). Reducing equivalents produced by substrate metabolism are transferred to the ETC., driving proton pumping across the inner mitochondrial membrane and establishing the electrochemical gradient (ΔΨm) (Busch, 2020; Javadov et al., 2021; Lapuente-Brun et al., 2013). This proton motive force is then harnessed by ATP synthase to phosphorylate ADP to ATP (Hong and Pedersen, 2008). Mitochondrial efficiency depends not only on the integrity of ETC complexes but also on an adequate supply of substrates, cofactors, and oxygen (Khalimonchuk and Rödel, 2005).

Beyond energy generation, mitochondria integrate calcium handling, ROS signaling, and dynamic remodeling processes such as fusion, fission, and mitophagy (Vásquez‐Trincado et al., 2016). These functions ensure not only efficient energy conversion but also quality control of the organelle population (Scheffer et al., 2022). The balance between ROS production and antioxidant defense is crucial, as physiological ROS mediate signaling whereas excess ROS contribute to oxidative injury (D’Oria et al., 2020). Clinical studies emphasize the physiological efficiency of mitochondria in the healthy heart. Measurements of high-energy phosphate flux by phosphorus-31 magnetic resonance spectroscopy (^31^P-MRS) show rapid ATP turnover, while human myocardial biopsies confirm intact ultrastructure and ETC activity (Peterzan et al., 2020; Rodgers et al., 2014). Such findings highlight why mitochondrial integrity is central to normal cardiac physiology.

### 2.2 Fatty acid oxidation: the primary energy source of the heart

Under normal physiological conditions, FAO is the main pathway through which the heart acquires energy, providing about 60%–90% of the myocardial energy demand. Long-chain fatty acids are activated to acyl-CoA in the cytosol, transported into mitochondria via the carnitine shuttle, and undergo sequential β-oxidation to generate acetyl-CoA, nicotinamide adenine dinucleotide hydride (NADH), and reduced flavin adenine dinucleotide (FADH_2_). These products then feed into the tricarboxylic acid (TCA) cycle and ETC., ultimately driving OXPHOS (Lopaschuk et al., 2010). Compared with glucose molecules, fatty acid molecules contain more carbon atoms and produce a significantly greater amount of ATP upon complete oxidation. One molecule of palmitic acid can theoretically generate up to 106 molecules of ATP, while one molecule of glucose under aerobic conditions yields a maximum of 32 ATP molecules (Jain et al., 2021). This high energy output efficiency enables the heart to meet its substantial and continuous energy needs. Besides, FAO produces relatively simple metabolic end products—primarily carbon dioxide and water—without generating large quantities of intermediate metabolites such as lactate, which are common in glucose metabolism (Wedan et al., 2024). This reduces disruption to intracellular acid–base homeostasis and supports the maintenance of a stable internal environment within cardiomyocytes.

**FIGURE 1:**
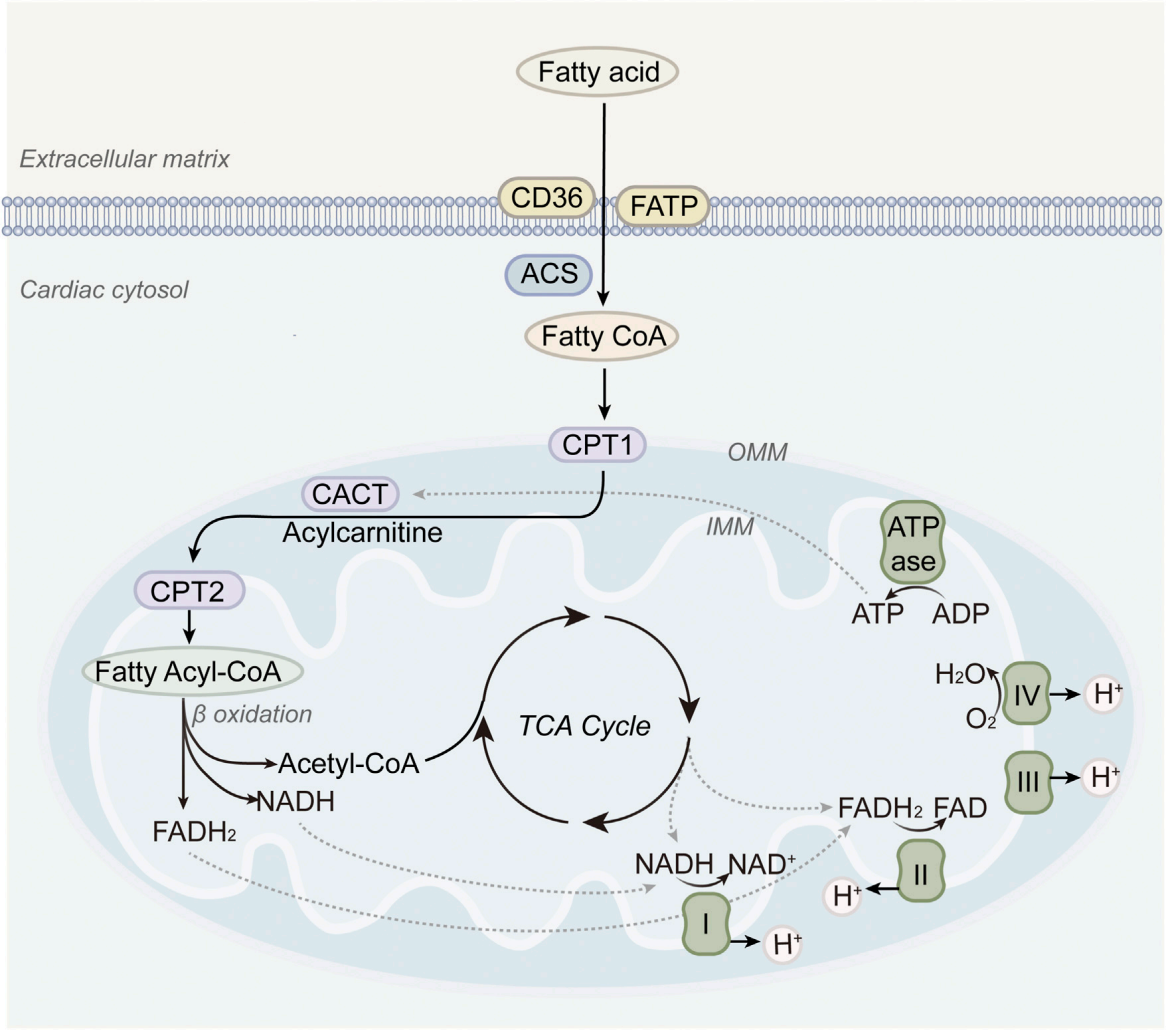
Fatty acid metabolism in normal hearts. Long-chain fatty acids are transported into mitochondria via CPT1, undergo β-oxidation, and feed into the TCA cycle and electron transport chain, thereby providing the majority of ATP required for cardiac contraction.

Clinical evidence strongly supports the central role of FAO in myocardial energetics. Positron emission tomography imaging demonstrates high rates of fatty acid uptake and oxidation in healthy human hearts (Sochor et al., 1986). In contrast, failing hearts often exhibit suppressed FAO accompanied by a compensatory increase in glucose utilization (Taylor et al., 2001). Despite its dominant role, it is increasingly recognized that excessive reliance on FAO may contribute to disease progression in HF. Impaired mitochondrial oxidative capacity can lead to incomplete FAO, energetic inefficiency, and toxic metabolite accumulation (Li et al., 2014; Lionetti et al., 2011). Therapies that partially inhibit FAO or enhance substrate flexibility, such as trimetazidine or perhexiline, have shown benefits in small clinical trials, suggesting that modulation rather than maximization of FAO may be the optimal strategy (Lee et al., 2005; Tuunanen et al., 2008; Fra et al., 2006). These concepts are illustrated in Figure 1, which provides an overview of fatty acid oxidation in the healthy heart.

### 2.3 The synergistic relationship between fatty acid oxidation and mitochondrial function in the normal heart

In healthy hearts, a coordinated relationship exists between FAO and mitochondrial function (Actis et al., 2024). Mitochondria serve as the vital site for FAO by containing the enzymatic machinery required for this process, while FAO, in turn, provides abundant substrates to support mitochondrial OXPHOS. These processes are tightly interdependent. The transport proteins and enzymes on the inner mitochondrial membrane, such as carnitine palmitoyltransferase 1 (CPT1) and the carnitine-acylcarnitine translocase (CACT), are critical for mitochondrial uptake and subsequent oxidation of fatty acids (Schlaepfer and Joshi, 2020; Grevengoed et al., 2014). Among them, CPT1 activity plays a rate-limiting role in the translocation of fatty acids into the mitochondrial matrix, thus directly regulating FAO flux.

When cardiac energy demand increases, neurohumoral regulatory mechanisms boost fatty acid uptake and enhance the activity of enzymes like CPT1, speeding up FAO to meet the increased metabolic needs. The reducing equivalents generated by FAO, namely, NADH and FADH_2_, serve as essential electron donors for the ETC., driving ATP synthesis through ATP synthase. Acetyl-CoA derived from FAO not only fuels the TCA cycle to support additional ATP production but also contributes to the synthesis of mitochondrial phospholipids such as cardiolipin, which is indispensable for maintaining inner membrane integrity and respiratory efficiency. Furthermore, mitochondrial ATP production supports energy-demanding steps within FAO, such as fatty acid activation. This synergistic interaction ensures that the heart can maintain efficient energy metabolism and sustain normal physiological function under varying metabolic states. Disruption of this coordination, whether through mitochondrial dysfunction leading to decreased CPT1 activity or insufficient substrate supply for FAO, can cause energy imbalance in the myocardium. Such impairments ultimately compromise both systolic and diastolic performance and may accelerate the onset and progression of heart failure.

## 3 Mitochondrial dysfunction in a failing heart

**FIGURE 2:**
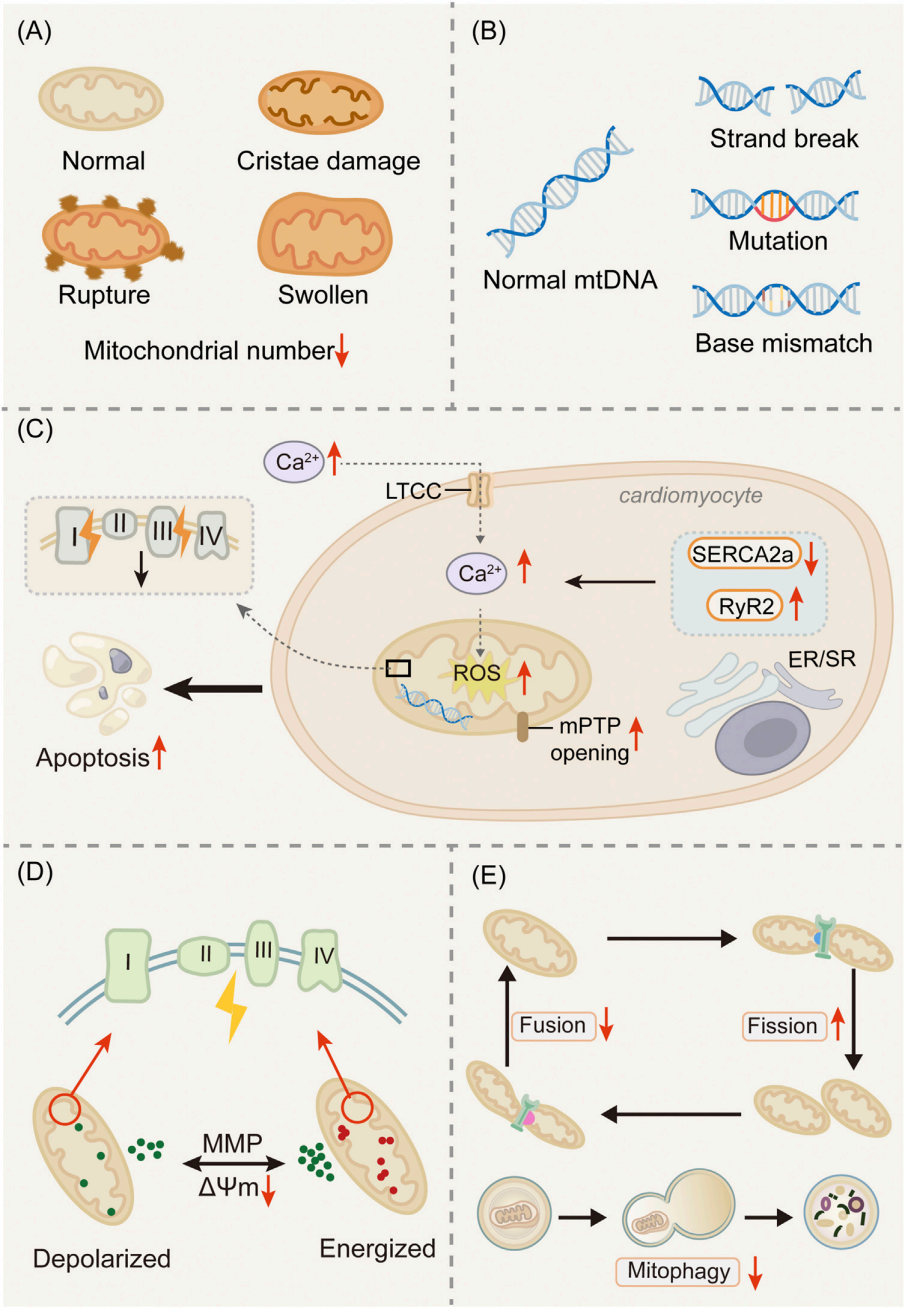
Mitochondrial dysfunction in the failing heart. **(A)** Mitochondrial alterations. **(B)** mtDNA mutations. **(C)** Oxidative stress and Ca^2+^ overload. **(D)** ΔΨm ombalance. **(E)** Disrupted mitochondrial dynamics. Damaged mitochondria exhibit cristae disruption, outer membrane rupture, and swelling, leading to impaired oxidative phosphorylation and reduced ATP production. Concomitantly, accumulation of mitochondrial DNA (mtDNA) mutations, increased reactive oxygen species (ROS) generation, and calcium overload exacerbate dysfunction. Dysregulation of mitochondrial dynamics (excessive fission, reduced fusion) and impaired mitophagy contribute to the persistence of defective organelles. Collectively, these abnormalities compromise bioenergetics and accelerate disease progression.

During the development and progression of HF, mitochondria undergo significant structural and functional alterations that profoundly affect myocardial energy metabolism and overall cardiac function (Figure 2).

### 3.1 Mitochondrial alterations

A reduction in the number of mitochondria is one of the key structural changes seen in heart failure (Rosca and Hoppel, 2010). Electron microscopy of myocardial sections revealed a dense and organized distribution of mitochondria in normal cardiomyocytes. In contrast, failing hearts exhibit a substantial decrease in mitochondrial density, leading to an insufficient number of “energy factories” to fulfill the high metabolic demands of cardiomyocytes, ultimately impairing cardiac function (Lesnefsky et al., 2018; Hinton et al., 2024). Besides quantitative changes, mitochondrial morphological abnormalities are also significant features of heart failure (Coluccia et al., 2018). In HF, mitochondria exhibit notable structural disruptions, including swelling, cristae fragmentation, loss or disappearance of cristae, and blurred or disrupted outer and inner membranes (Daghistani et al., 2019). Cristae remodeling is significant, as cristae architecture determines the organization of the ETC supercomplexes. Disrupted cristae reduce ETC efficiency and increase electron leakage, promoting ROS generation. Cardiolipin depletion or oxidation, frequently observed in HF, provides a molecular explanation for these defects. Still, whether such structural changes are a primary driver of dysfunction or a secondary response remains unresolved (Collins et al., 2021; Ozcan et al., 2003).

Endomyocardial biopsies from HF patients show swollen mitochondria and cristae disruption, correlating with reduced ejection fraction. Pediatric HF exhibits similar changes, indicating that these alterations are not limited to adult or ischemic disease (Nakano et al., 2024; Pawlak et al., 2022). Nevertheless, inconsistencies remain. Some experimental models report preserved mitochondrial density even in advanced HF, and it is unclear whether structural injury precedes or follows contractile dysfunction. In summary, structural and ultrastructural mitochondrial abnormalities are closely associated with impaired energetics and adverse outcomes in HF. Yet their heterogeneity and uncertain causality highlight the need for integrated clinical and translational studies to clarify whether mitochondrial remodeling represents a therapeutic target or primarily a disease marker.

### 3.2 Mitochondrial DNA mutations

Mitochondrial DNA (mtDNA) is particularly vulnerable to injury in the failing heart (Garcia et al., 2017). Unlike nuclear DNA, mtDNA lacks protective histones and is located near the electron transport chain, where ROS are continuously generated (Chen and Zweier, 2014). As a result, mtDNA accumulates mutations and deletions that compromise the synthesis of respiratory chain proteins encoded by mitochondrial genes. Such defects impair OXPHOS, reduce ATP production, and exacerbate ROS generation, forming a self-perpetuating cycle of mitochondrial injury (Nakayama and Otsu, 2018).

Multiple studies have identified mtDNA deletions and point mutations in the myocardium of HF patients. These abnormalities correlate with decreased activity of respiratory complexes I and IV, which depend heavily on mtDNA-encoded subunits. Large-scale cohort analyses, such as the Framingham Heart Study, have reported that the myocardial mtDNA mutation burden in HF patients is approximately 3.5 times higher than in age-matched controls (Ide et al., 2001). Among these, the 4977-bp “common deletion” was detected in 27% of HF patients compared with only 2% in controls (Wei et al., 2014). Increased levels of circulating cell-free mtDNA fragments have also been detected in HF, suggesting that mtDNA damage contributes not only to bioenergetic decline but also to pro-inflammatory signaling through activation of innate immune pathways (Oka et al., 2012; West and Shadel, 2017). In endomyocardial biopsies, higher burdens of mtDNA mutations are associated with reduced ejection fraction and worse outcomes (Gallardo et al., 2012). Circulating mtDNA levels have been proposed as potential biomarkers of disease severity and prognosis, though validation in larger cohorts is needed. Importantly, not all studies agree on the extent of mtDNA mutations in HF, and some discrepancies may reflect differences in patient populations, sequencing approaches, or disease stage (Gustafsson and Dorn, 2019). Taken together, mtDNA mutations link oxidative stress, energetic failure, and inflammation in HF, and both myocardial and circulating mtDNA profiles may serve as valuable diagnostic and prognostic tools. However, mechanistic and longitudinal clinical studies are needed to establish causality and therapeutic potential.

### 3.3 Oxidative stress and calcium overload

Oxidative stress and calcium overload are tightly interconnected mechanisms of mitochondrial dysfunction in HF. Excessive ROS generated by the electron transport chain damages mitochondrial membranes, proteins, and DNA, while impaired calcium handling increases cytosolic and mitochondrial Calcium (Ca^2+)^ levels, further compromising bioenergetics (Dridi et al., 2023). ROS and calcium form a self-reinforcing cycle. ROS facilitates the opening of the mitochondrial permeability transition pore (mPTP), leading to swelling and membrane depolarization. In turn, Ca^2+^ overload enhances ROS production by stimulating dehydrogenases and destabilizing ETC function. Disruption of mitochondrial–sarcoplasmic reticulum (SR) crosstalk exacerbates this cycle, weakening excitation–contraction coupling and reducing contractile reserve (Zorov et al., 2014; Bertero and Maack, 2018).

These abnormalities have direct consequences for myocardial energy metabolism. Under physiological conditions, mitochondrial Ca^2+^ uptake activates dehydrogenases in the TCA cycle, enhancing NADH generation and ATP production. In failing hearts, however, Ca^2+^ overload surpasses the regulatory threshold, triggering mPTP opening and ATP loss. Similarly, ROS-mediated damage to ETC complexes decreases OXPHOS efficiency, forcing the heart to rely on less efficient metabolic pathways (Morad and Suzuki, 2000). Oxidative modifications of key enzymes, such as pyruvate dehydrogenase and fatty acyl-CoA dehydrogenase, further impair substrate utilization. These disruptions explain, at least in part, the consistently observed decline in the phosphocreatine-to-ATP ratio in HF patients.

In myocardial samples from HF patients, glutathione peroxidase (GPx) activity is reduced by 52% compared with controls, and this reduction is inversely correlated with elevated plasma levels of 8-isoprostanes, a marker of oxidative stress (Mallat et al., 1998). These findings suggest that impaired antioxidant defenses are directly associated with systemic oxidative stress in patients. On the calcium side, altered expression of calcium-handling proteins, such as reduced sarcoplasmic reticulum Ca^2+^ ATPase(SERCA2a) and dysfunctional ryanodine receptors, has been documented in failing human hearts, providing a mechanistic basis for impaired excitation–contraction coupling (Hasenfuss et al., 1994). Moreover, the CUPID II trial showed that SERCA2a gene transfer, although failing to improve primary endpoints, reduced abnormal calcium transients, suggesting that more refined approaches may still hold potential (Jessup et al., 2011). In summary, oxidative stress and calcium overload not only damage mitochondrial structure but also destabilize myocardial energy metabolism. Their intertwined effects—impairing OXPHOS, disrupting substrate utilization, and lowering ATP availability—highlight why HF is fundamentally an energetic disease. Future therapeutic strategies should aim to restore redox balance and calcium homeostasis in a manner that also optimizes metabolic efficiency.

### 3.4 Mitochondrial membrane potential imbalance

In the context of HF, the ΔΨm—a critical bioelectrical gradient essential for maintaining mitochondrial function and myocardial energy metabolism—is significantly disrupted (Briston et al., 2017). In healthy hearts, ΔΨm drives OXPHOS, ensuring efficient coupling of electron transport to ATP production (Garbincius and Elrod, 2022). In HF, however, ΔΨm becomes destabilized, leading to impaired ATP generation and increased susceptibility to cell death (Brown et al., 2017; Koulintchenko et al., 2003; Schneider, 2011; Martínez-Reyes et al., 2016). Studies have shown that ΔΨm reduction in failing cardiomyocytes is associated with defective ETC activity, impaired fatty acid oxidation, and lower efficiency of oxidative phosphorylation. Conversely, an excessively high ΔΨm can enhance electron leakage and promote ROS generation, suggesting that ΔΨm must be maintained within an optimal range. This dual role highlights ΔΨm as both a marker and a potential modulator of bioenergetic balance.

Myocardial biopsy samples from patients with dilated cardiomyopathy demonstrate reduced ΔΨm together with diminished complex I and IV activities. Elevated circulating mtDNA and ROS markers in HF have also been associated with impaired mitochondrial polarization, supporting the role of ΔΨm imbalance as a systemic phenomenon (Di Lisa et al., 1995; Zorov et al., 1992). Despite these findings, several questions remain. It is not clear whether ΔΨm collapse is a primary driver of contractile dysfunction or a secondary consequence of metabolic stress. ΔΨm imbalance in HF disrupts the delicate balance between ATP synthesis and ROS production, linking mitochondrial bioenergetics to clinical outcomes. While experimental evidence is compelling, further translational studies are needed to clarify whether ΔΨm is best considered a therapeutic target or a biomarker of disease severity.

### 3.5 Disrupted mitochondrial dynamics

Mitochondrial dynamics, which include the processes of mitochondrial fusion and fission, are essential for maintaining structural integrity, responding to metabolic demands, and eliminating damaged mitochondria (Tokuyama and Yanagi, 2023). Fusion proteins such as mitofusin 1/2 (Mfn1/2) and optic atrophy 1 (OPA1) enable mitochondrial content mixing and preserve OXPHOS efficiency (Santel and Fuller, 2001; Hu et al., 2022), whereas fission proteins like dynamin-related protein 1 (Drp1) facilitate organelle segregation and turnover (Hu et al., 2020). Mitophagy, primarily mediated by the PTEN-induced kinase 1 (PINK1) and Parkin RBR E3 ubiquitin-protein ligase (Parkin) pathway, eliminates dysfunctional mitochondria, thereby preventing excessive ROS accumulation and sustaining ATP production. In HF, this balance is disrupted. Excessive fission and reduced fusion result in mitochondrial fragmentation, impaired assembly of ETC supercomplexes, and decreased ATP synthesis (Xu et al., 2023). Defective mitophagy allows damaged mitochondria to accumulate, aggravating oxidative stress and metabolic inflexibility. These abnormalities directly compromise fatty acid oxidation and TCA cycle activity, contributing to energy failure (Chen et al., 2005).

Myocardial biopsies from HF patients reveal upregulation of Drp1 and downregulation of Mfn2, findings that correlate with reduced left ventricular ejection fraction (Chen et al., 2009; Song et al., 2015). Circulating levels of mitophagy-related proteins, including PINK1, have been proposed as non-invasive markers of disease severity (Bil et al., 2011; Shirakabe et al., 2016). Abnormal mitochondrial dynamics in HF disrupt energetic efficiency by impairing fusion, fission, and mitophagy. While both experimental and clinical data highlight their relevance, therapeutic translation remains limited, warranting larger trials to determine whether restoring mitochondrial dynamics can meaningfully improve patient outcomes.

## 4 Interplay between mitochondrial dysfunction and fatty acid metabolic disturbances in the failing heart

**FIGURE 3:**
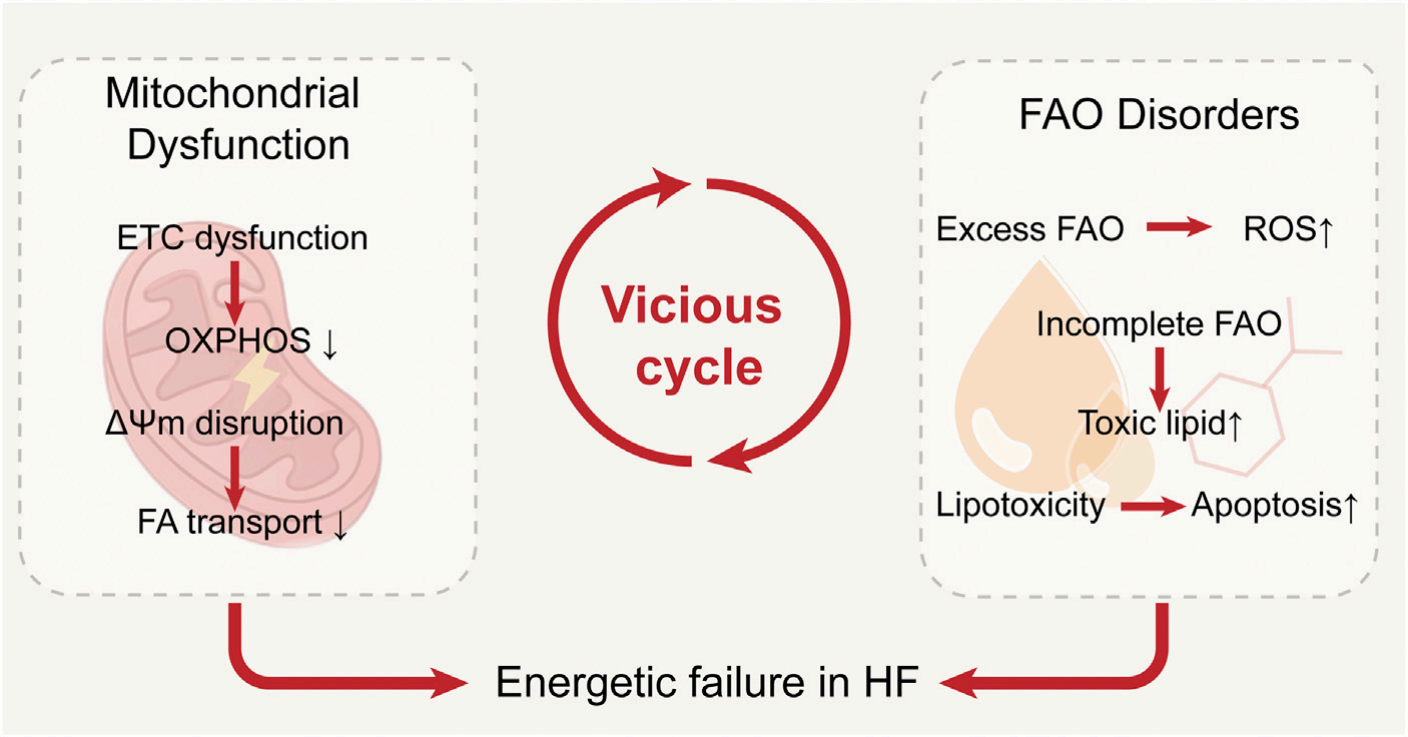
Interplay between mitochondrial dysfunction and fatty acid metabolic disturbances in the failing heart. Impaired oxidative phosphorylation reduces fatty acid oxidation and promotes lipid accumulation, while excessive fatty acid uptake and incomplete β-oxidation aggravate mitochondrial damage through ROS production, together forming a vicious cycle that accelerates heart failure progression.

Mitochondrial dysfunction and FAO disorders reinforce each other in the failing heart: ETC defects and ΔΨm loss suppress FAO, while dysregulated FAO promotes oxidative stress, lipotoxicity, and structural damage, further compromising mitochondrial function. This vicious cycle of energetic failure not only drives disease progression but also reveals potential therapeutic targets. These interactions are summarized in Figure 3.

### 4.1 ETC dysfunction suppresses FAO

FAO is the predominant energy source for the healthy adult heart, but its efficiency relies heavily on intact ETC function. In HF, ETC dysfunction directly compromises FAO by limiting the regeneration of NAD^+^ and FAD, which are required for β-oxidation. Reduced electron flow through complexes I and III slows downstream OXPHOS, leading to substrate accumulation and impaired metabolic flexibility (Korepanov et al., 2024). Myocardial biopsies from HF patients demonstrate a 40% reduction in complex I activity, which correlates strongly with decreased CPT1a expression (Karam et al., 2013). Metabolomic profiling further reveals accumulation of long-chain acylcarnitines in plasma, and their levels are inversely correlated with left ventricular ejection fraction (LVEF) (Chokshi et al., 2012; Cheng et al., 2015). These findings suggest that ETC dysfunction impedes FAO not only at the enzymatic level but also at the systemic metabolic level, contributing to cardiac energetic failure.

AMP-activated protein kinase (AMPK) plays a critical but context-dependent role in this process (Qi and Young, 2015). In the early stages of HF, AMPK activation enhances glucose uptake and promotes metabolic flexibility, thereby exerting protective effects. However, in advanced HF, persistent AMPK activation has been associated with transcriptional suppression of CPT1, accelerating the decline in FAO capacity and reinforcing metabolic rigidity (Cantó et al., 2009). This paradox highlights the stage-dependent duality of AMPK signaling: adaptive in early disease but maladaptive in later stages, as documented in recent clinical evidence (Wang et al., 2009). Together, these data underscore that ETC dysfunction not only impairs ATP production directly but also indirectly suppresses FAO, exacerbating energetic insufficiency. The interplay between ETC activity, FAO regulation, and AMPK signaling suggests that therapeutic approaches must account for disease stage. Interventions aimed at restoring ETC function or selectively modulating AMPK activity may help preserve metabolic flexibility, though further clinical validation is needed.

### 4.2 ΔΨm disruption hinders FA transport

During the progression of HF, the stability of the ΔΨm is essential for facilitating the transmembrane transport and oxidation of fatty acids. ΔΨm is generated by the proton-pumping activity of ETC Complexes I, III, and IV and functions as a crucial energy source for many transporters within the inner mitochondrial membrane. The exchange of long-chain acylcarnitines into the mitochondrial matrix via CACT, although electrically neutral, still depends on a stable ΔΨm to maintain the proton gradient across the inner and outer mitochondrial membranes and to support the overall translocation driving force (Martínez-Reyes et al., 2016).

In HF, collapse of ΔΨm reduces the driving force for acylcarnitine exchange, leading to impaired mitochondrial fatty acid uptake and oxidation (Wende and Abel, 2010). This deficit in fatty acid transport contributes to substrate accumulation in the cytosol, promoting lipotoxicity. Excess long-chain acylcarnitines and ceramides disrupt sarcolemmal integrity, trigger arrhythmogenic signaling, and exacerbate contractile dysfunction. Importantly, these lipid intermediates further destabilize mitochondrial membranes and aggravate ΔΨm loss, forming a pathological feedback loop that perpetuates energetic failure. In HF patients, reduced ΔΨm has been associated with accumulation of plasma long-chain acylcarnitines and impaired myocardial ATP synthesis. These findings indicate that ΔΨm imbalance not only disrupts oxidative phosphorylation but also alters substrate flux, linking mitochondrial bioenergetics to systemic metabolic signatures.

### 4.3 FAO disorders impair mitochondrial function

Disordered FAO not only reflects mitochondrial dysfunction but also feeds back to further impair mitochondrial integrity and bioenergetics. Excessive reliance on FAO produces large amounts of NADH and FADH_2_, which overload the ETC and increase electron leakage. This results in exacerbated oxidative stress, as demonstrated by elevated malondialdehyde and 8-isoprostane levels in HF patients, along with reduced myocardial antioxidant enzyme activity (Ide et al., 2001; Mallat et al., 1998). Such oxidative injury destabilizes mitochondrial membranes and impairs OXPHOS.

In addition, incomplete FAO leads to accumulation of toxic lipid intermediates. Long-chain acylcarnitines, diacylglycerol, and ceramide have been detected at elevated levels in both plasma and myocardial tissue of HF patients, where they correlate inversely with LVEF (Bedi et al., 2016). These metabolites impair ΔΨm, promote opening of the mPTP, and activate pro-apoptotic signaling, thereby amplifying mitochondrial injury. Importantly, these lipotoxic intermediates also disrupt calcium handling, aggravating excitation–contraction uncoupling and energetic inefficiency (Alevriadou et al., 2021).

Finally, persistent FAO dysregulation contributes to structural mitochondrial damage and impaired ATP production. Human myocardial biopsies reveal swollen, fragmented mitochondria with disrupted cristae in advanced HF, accompanied by reduced phosphocreatine-to-ATP ratios (Karam et al., 2013; Neubauer, 2007). These findings demonstrate that FAO disorders directly impair mitochondrial architecture and energy coupling in the failing heart.

These insights have motivated the use of partial FAO inhibitors such as trimetazidine (TMZ) and perhexiline, which improve exercise tolerance and symptoms in HF patients by reducing lipotoxic accumulation and enhancing glucose utilization (Lee et al., 2005; (Mouquet et al., 2010). However, these agents have not consistently improved survival, highlighting the complexity of FAO–mitochondrial interactions and the limitations of targeting FAO in isolation. Future strategies should aim at restoring balanced substrate utilization and protecting mitochondrial structure, rather than pursuing uniform suppression of FAO. FAO disorders impair mitochondrial function through oxidative stress, accumulation of toxic lipid intermediates, and structural disruption. This negative feedback loop underscores the dual role of FAO in sustaining and simultaneously undermining cardiac energetics, with important implications for therapeutic modulation.

### 4.4 Limitations and future directions

While the bidirectional interplay between mitochondrial dysfunction and FAO disorders provides a compelling conceptual framework, it should be emphasized that direct mechanistic evidence, particularly in humans, remains limited. Much of the supporting data derives from animal models or correlative clinical observations, and therefore, this concept must be interpreted with caution (Rosca and Hoppel, 2010; Doenst et al., 2013). Distinguishing between well-supported mechanisms and speculative links is essential to avoid overstating the strength of current evidence.

Although considerable progress has been made in understanding the interplay between mitochondrial dysfunction and FAO disorders in HF, several limitations remain. First, much of the mechanistic evidence derives from animal or cell-based studies, whereas human data are comparatively scarce and often limited to small cohorts or end-stage HF biopsies (Lopaschuk et al., 2010). This raises concerns about generalizability across disease etiologies and stages. Moreover, current evidence frequently provides correlative rather than causal insights. For instance, elevated plasma acylcarnitines and reduced PCr/ATP ratios are robust markers of HF severity, yet whether they directly drive progression or primarily reflect secondary metabolic stress is still debated (Cheng et al., 2015; Neubauer, 2007). Similarly, the extent to which mitochondrial ultrastructural damage is a cause or consequence of energetic failure remains unresolved (Karam et al., 2013).

Looking forward, future research should prioritize large-scale, longitudinal studies that incorporate multi-omics profiling (transcriptomics, metabolomics, and imaging) to define patient-specific metabolic signatures (Adams et al., 2018). Such approaches may enable better stratification of HF subtypes and identification of individuals most likely to benefit from metabolic therapies. Novel pharmacological strategies may include dual-target approaches, like partial FAO inhibition plus glucose or ketone promotion, interventions that enhance mitophagy or preserve mitochondrial DNA integrity, and precision therapies tailored to disease stage (Karam et al., 2013). Integrating mechanistic insights with clinical validation will be essential to move beyond descriptive correlations toward effective metabolic interventions.

## 5 Therapeutic strategies targeting mitochondrial and fatty acid metabolism

Pharmacological agents, natural products, traditional herbal formulations, gene and cell therapies, and lifestyle interventions have all been explored for their ability to restore mitochondrial function, optimize FAO, and improve cardiac energy homeostasis. Growing clinical evidence supports the translational relevance of these strategies, though the strength of evidence varies across approaches.

### 5.1 Pharmacological agents

SGLT2 inhibitors exemplify the most clinically validated metabolic therapy. Beyond their glucose-lowering effects, dapagliflozin and empagliflozin enhance myocardial energy efficiency by promoting ketone body oxidation while preserving FAO, mitigating substrate imbalance. Mechanistic studies indicate an increased mitochondrial NAD^+^/NADH ratio, improved electron transport chain efficiency, and reduced ROS accumulation (Goedeke et al., 2024; Croteau et al., 2021; Chambers et al., 2025). Large-scale randomized trials in both diabetic and non-diabetic HF populations consistently show reduced hospitalization, improved LVEF, and better quality of life, confirming the clinical relevance of targeting cardiac metabolism (Santos-Gallegoet al., 2019; Su et al., 2023).

TMZ shifts myocardial substrate utilization toward glucose oxidation, increasing ATP production efficiency. Preclinical studies show reduced myocardial lipid accumulation, oxidative stress, and apoptosis, with preserved mitochondrial membrane potential and calcium homeostasis (Kantor et al., 2000; Li et al., 2020). Clinically, TMZ improves systolic function and exercise tolerance without adverse hemodynamic effects, highlighting its potential as an adjunct therapy, especially in patients with impaired FAO (Fragasso et al., 2011).

Glucagon-like peptide-1 receptor agonists (GLP-1RAs), such as semaglutide, enhance pyruvate entry into the TCA cycle and promote FAO, reducing lipid accumulation, preserving mitochondrial function, and attenuating cardiac remodeling in experimental models (Ma et al., 2024). Cardiovascular outcome studies in diabetic populations suggest potential cardioprotective effects, though dedicated HF trials are needed.

Other pharmacological strategies include β-adrenergic receptor agonists and pan-estrogen-related receptor (ERR) agonists. In diabetic HF models, β-adrenergic stimulation maintains metabolic flexibility, balancing FAO and glucose oxidation (Lindsay et al., 2024). ERR agonists, including SLU-PP-332 and SLU-PP-915, enhance mitochondrial oxidative capacity, promote FAO, reduce fibrosis, and preserve contractile function in preclinical studies, suggesting potential targeted metabolic therapies (Xu et al., 2024a).

Mitochondrial protectants like coenzyme Q10 (CoQ10) support electron transport and ATP production (Pei et al., 2022; Sanbe et al., 1994). Meta-analyses demonstrate improvements in LVEF and New York Heart Association (NYHA) class, although low bioavailability limits clinical impact, emphasizing the need for optimized formulations or combination strategies (Fumagalli et al., 2011). Overall, pharmacological modulation of cardiac metabolism is clinically feasible, with SGLT2 inhibitors leading the field and other agents showing promise pending further validation (Table 1).

**TABLE 1:** Pharmacological agents targeting mitochondrial function and fatty acid metabolism in HF.

Agent	Models	Effectiveness indicators and pathways	Effect of action	Refer
SGLT2i	1) Gαq-overexpressing male mice 2) Ligation of the LAD-induced HF in rats 3) Non-diabetic ischemic HF rat model	1) mTOR signaling pathway↓ 2) AMPK/SIRT-1/PGC-1α signaling pathway↑ 3) PPARα↑	1) Prevented the development of apoptosis, myocyte hypertrophy and fibrosis 2) Prevented mitochondrial dysfunction and oxidative stress	Goedeke et al. (2024), Croteau et al. (2021), Chambers et al. (2025)
Trimetazidine	ISO-induced HF rats	1) AMPK/PPARα signaling pathway↑ 2) GLUT 4↑, CPT 1↑, 3-KAT↓, BDH1↑, ACAT1↑, HMGCS2↑, MCT1↑	1) Improved cardiac function 2) Attenuated metabolic remodeling	Kantor et al. (2000), Li et al. (2020)
Semaglutide	TAC surgical in rats	1) Creb5/NR4a1 signaling pathway↓ 2) PI3K/AKT signaling pathway↓ 3) Drp1↓, Opa1↑, Mfn2↑, SDHB↑, NDUFV2↑, ATP5A1↑	1) Improved hypertrophy, fibrosis and contractility Modulated axis 2) Improved mitochondrial morphology and function 3) Reduced glycolysis and free fatty acid accumulation	Ma et al. (2024)
Isoprenaline	90% Px in diabetic rats	1) cGMP↑, CD36↑, CPT-1↑, GLUT1↑, PDK1↓, PDK2↓	1) Maintained FAO/glucose balance 2) Promoted cardiac mitochondrial respiratory capacity	Lindsay et al. (2024)
SLU-PP-332/915	TAC-induced in HF mice	1) ERK 1/2 signaling pathway↑ 2) NFAT↑, ACACB↑, ACADM↑, ACSL1↑, CPT1b↑	1) Improved cardiac function 2) Reduced cardiac fibrosis 3) Ameliorated mitochondrial dysfunction	Xu et al. (2024a)

### 5.2 Exploration of herbal medicine-based therapies

Many TCM fomulas and natural compounds have demonstrated unique effects in improving mitochondrial function and regulating fatty acid metabolism, providing novel therapeutic strategies for the management of HF.

#### 5.2.1 Natural products

Natural products have garnered increasing attention for their multitarget regulatory potential in HF, particularly through improving mitochondrial function and restoring fatty acid metabolism. Neocryptotanshinone (NCTS), a bioactive constituent of *Salvia miltiorrhiza* (Danshen), exhibits antioxidant, anti-inflammatory, and antiapoptotic properties. Mechanistic studies indicate that NCTS enhances FAO by upregulating retinoid X receptor alpha (RXRα)/peroxisome proliferators activated receptors (PPARα) downstream genes and promotes mitochondrial biogenesis via recombinant transcription factor A (TFAM), resulting in elevated myocardial ATP levels and mitigated ischemic injury (Ma et al., 2023). Clinically, small trials using Danshen extracts have reported improvements in cardiac function and exercise tolerance (Chen et al., 2017). However, sample sizes are limited and endpoints are heterogeneous, highlighting the need for larger, well-designed studies.

Cinnamaldehyde (CIN), the primary active compound in *Cinnamomum cassia*, has antioxidant, anti-inflammatory, and antifibrotic effects. It delays the progression of HF by inhibiting myocardial fibrosis, reducing oxidative stress, and suppressing the Transforming growth factor-β(TGF-β)/Smad signaling pathway. Moreover, CIN directly binds to and inhibits G protein-coupled receptor kinase 2 (GRK2), promoting its ubiquitination and degradation (Xu et al., 2024b). This activates the AMPK/Peroxisome proliferator-activated receptor γ coactivator 1-α(PGC-1α) pathway, restores disrupted fatty acid metabolism in cardiomyocytes, maintains mitochondrial energy homeostasis, and suppresses myocardial fibrosis and hypertrophy. Cinnamon-derived compounds have shown modest improvements in surrogate markers of cardiovascular function, such as N-terminal pro-brain natriuretic peptide (NT-proBNP) and echocardiographic indices, suggesting potential translational relevance, though rigorous HF trials are still lacking.

Extracts from *Artemisia argyi* (AA) contain various flavonoids and volatile oils and exert cardioprotective effects through anti-inflammatory, antioxidant, and anti-apoptotic mechanisms (Chen et al., 2024). In doxorubicin-induced cardiomyopathy models, AA extract significantly preserved cardiac function, reducing mitochondrial fission and oxidative stress, and maintaining cellular structure and energy production. While clinical evidence is scarce, AA-based therapies have been traditionally used to support cardiovascular health, and early pilot studies suggest potential benefit in preserving cardiac function.

Astragaloside IV (AS-IV), the principal active component of *Astragalus membranaceus,* exhibits antifibrotic, anti-apoptotic, antioxidant, and energy metabolism-enhancing properties in cardiomyocytes (Wang et al., 2024). It has been demonstrated to inhibit myocardial remodeling and improve cardiac systolic function by targeting the TGF-β1/Smad, Phosphatidylinositol 3-kinase (PI3K)/Protein Kinase B (AKT), and Nuclear Factor kappa-B(NF-κB) signaling pathways. AS-IV also decreases serum levels of proinflammatory cytokines. Additionally, by activating the AMPK/PGC-1α pathway, AS-IV enhances myocardial glucose and fatty acid metabolism and influences metabolic reprogramming and inflammation, thereby reducing myocardial fibrosis and improving cardiac function. Clinical studies have shown AS-IV-containing formulations can improve LVEF and exercise capacity in HF patients (Zhou et al., 2001), providing preliminary evidence for translational potential.

Ginsenoside Rb3 (G-Rb3), one of the major saponins in *Panax ginseng*, enhances FAO and mitochondrial function via PPARα activation and upregulation of key β-oxidation enzymes and Sirtuin 3(SIRT3) (Chen et al., 2019). Preclinical studies show improved energy metabolism, reduced apoptosis, and preserved cardiac structure. Limited clinical trials using ginseng extracts report improved functional capacity and reduced cardiac biomarkers (Yang et al., 2022), though standardized dosing and large-scale randomized controlled trials (RCTs) are needed to confirm efficacy.

Collectively, these natural products demonstrate multitarget modulation of HF-related metabolic pathways. While preclinical data are compelling, clinical translation remains limited by small sample sizes, heterogeneity in endpoints, and variations in compound standardization. These agents may be most promising as adjunctive therapies in patients with metabolic phenotypes characterized by mitochondrial dysfunction or FAO impairment (Table 2).

**TABLE 2:** Natural compounds targeting mitochondrial function and fatty acid metabolism in HF.

Natural product	Models	Effectiveness indicators and pathways	Effect of action	Refer
Neocryptotanshinone	1) Ligation of the LAD-induced post-MI HF in mice 2) OGD/R-induced H9c2 cells	1) RXRα/PPARα signaling pathway↑ 2) CD36↑, TFAM↑, CPT-1↑, NRF 1↑, Ppargc1a↑, CYTB↑	1) Improved cardiac function and structure 2) Increased myocardial ATP content 3) Decreased apoptosis and ROS production 4) Increased FAO and mitochondrial biogenesis	Ma et al. (2023)
Cinnamaldehyde	1) ISO-induced HF in rats 2) ISO-induced NRCMs	1) AMPK/PGC-1α signaling pathway↑ 2) GRK2↑, CD36↑, NRF 1↑, CPT-1B↑, ACADM↑	1) Improved cardiac function 2) Reduced myocardial hypertrophy and myocardial fibrosis 3) Improved FA metabolism and cardiac energy metabolism	Xu et al. (2024b)
Artemisia argyi	DOX-induced H9c2 cells	1) IGF-IIR/Drp1/GATA4 signaling pathway↓ 2) caspase 3↓, Akt↓, NFATC3↓, p-GATA4↓	1) Protected against DOX-induced cardiotoxicity 2) Reduced mitochondrial ROS 3)Increased hypertrophy and mitochondrial dysfunction	Chen et al. (2024)
Astragaloside IV	HFD + L-NAME induced HFpEF in mice	CRP↓, IL1RL1↓, GDF15↓, IL-1↓, IL-6↓, Caspase-1↓, NLRP3↓, VCAM-1↓	1) Improved cardiac function and structure 2) Suppressed inflammatory pathways 3)Improved mitochondrial function and glucose metabolism	Wang et al. (2024)
Ginsenoside Rb3	1) LAD ligation-induced HF in rats 2) OGD/R-induced H9C2 cells	1) FADD↓, PPARα↑ 2) ACADM↑, CD36↑, CPT-1B↑, CPT-1α↑, SIRT3↑, ACADL↑, Bcl-2↑, Bax↓	1) Improved cardiac function 2) Inhibited cardiac hypertrophy and fibrosis 3) Increased ATP generation 4) Protected mitochondrial function 5) Inhibited apoptosis	Chen et al. (2019)

#### 5.2.2 Traditional Chinese herbal formulas

Qiliqiangxin (QLQX) capsules, a traditional Chinese medicine composed of 11 herbal ingredients, have been shown to exert significant cardioprotective effects in various HF models, particularly through a unique ability to modulate myocardial energy metabolism (Cheng et al., 2020). QLQX promotes regional metabolic reprogramming, enhancing Glucose Transporter 4 (GLUT4)-mediated glucose uptake and activating anaerobic glycolysis in peri-infarct regions, while restoring FAO in remote myocardium via upregulation of CD36 and CPT-1 (Wang et al., 2020a). Additionally, QLQX activates PINK1/Parkin-mediated mitophagy, reducing apoptosis and preserving mitochondrial integrity, ultimately delaying cardiac remodeling (Zhou et al., 2020). Clinical studies, including randomized controlled trials in post-myocardial infarction HF patients, report improvements in LVEF, NT-proBNP, and NYHA functional class, supporting translational relevance (Li et al., 2013; Cheang et al., 2024).

Qishen granules (QSGs), another classic multiherbal formulation in traditional Chinese medicine, have been widely used in China for the treatment of cardiovascular diseases and have shown notable clinical efficacy. Multiple studies have demonstrated the QSG upregulates fatty acid transporters and β-oxidation enzymes, activates the PPARα/RXRα signaling axis, and modulates hypoxia-inducible factor (HIF-1α) pathways, effectively restoring energy metabolism disrupted by ischemia (Gao et al., 2020; Sun et al., 2025). Moreover, QSG has been shown to improve LVEF, reduce circulating lipid and lactate levels, and attenuate remodeling, confirming the formula’s systemic metabolic effects (Wang et al., 2017; Du et al., 2022). Notably, QSG may be particularly beneficial in ischemic HF patients with impaired FAO and mitochondrial dysfunction.

Shenmai injection (SMI) is a traditional Chinese herbal formulation based on the TCM principle of replenishing Qi and nourishing Yin. SMI, which is composed primarily of *P. ginseng* and *Ophiopogon japonicus*, has shown considerable therapeutic potential in cardiovascular diseases in recent years. Integrative pharmacology and experimental validation have identified 48 bioactive components in SMI that target the PPARα/SIRT1/PGC-1α signaling pathway (Yang et al., 2024). These compounds significantly reduce serum free fatty acid (FFA) levels, preserve mitochondrial morphology and function in cardiomyocytes, and thus attenuate myocardial fibrosis and apoptosis. In a mouse model of left anterior descending coronary artery ligation, SMI administration improved the ejection fraction and fractional shortening. It restored the ΔΨm in ischemia‒reperfusion-injured cells, supporting the role of SMI in metabolic reprogramming and energy restoration. Clinical efficacy has also been substantiated in randomized controlled trials. A single-masked study involving 120 HF patients reported significant improvements in NYHA class, BNP, LVEF, and metabolic biomarkers after SMI administration, with superior effects compared to trimetazidine, suggesting a synergistic advantage of multitarget interventions (Wang et al., 2020b).

Xinbao pill (XBP) is a classical TCM compound derived from modifications of the formulas “Liu Shen Wan” and “Shenfu Tang,” which contain nine herbal ingredients, including *P. ginseng*, *Panax notoginseng*, and *Aconitum carmichaeli*. It has been clinically applied in the treatment of HF. Recent studies in a rat model of isoprenaline-induced HF demonstrated that 6 weeks of XBP treatment significantly improved LVEF and left ventricular fractional shortening (LVFS), reduced myocardial hypertrophy and fibrosis, and mitigated mitochondrial damage (Pan et al., 2024). Mechanistic studies revealed that XBP exerts its effects by downregulating SGLT1, activating AMPK phosphorylation, and promoting the nuclear translocation and transcriptional activation of PPARα, thus increasing FAO and restoring the cardiac energy supply. Importantly, SGLT1 knockdown further enhanced the regulatory effects of XBP on myocardial energy metabolism, whereas SGLT1 overexpression reversed its cardioprotective effects, confirming that SGLT1 is a key molecular target in the improvement of XBP-mediated FAO.

Several formulations, including Qiliqiangxin and Shenmai injection, have already demonstrated efficacy in randomized clinical trials, highlighting their translational potential. However, larger, longer-term studies are required to confirm their impact on hard clinical outcomes and further validate their mechanisms of action in patients. Nonetheless, these interventions may offer valuable adjunctive options, particularly for patients with HF characterized by metabolic impairment or mitochondrial dysfunction (Table 3).

**TABLE 3:** TCM Formula targeting mitochondrial function and fatty acid metabolism in HF.

TCM formula	Models	Effectiveness indicators and pathways	Effect of action	Refer
Qiliqiangxin (QLQX)	1) Ligation of the LAD-induced HF in rats and mice 2) Hypoxia-induced H9c2 cells	1) Pink1/Parkin signaling pathway↑ 2) HIF-1α/VEGF signaling pathway↑ 3) CPT-1↑, CD 36↑, PDH↑, PDK4↑, GLUT1↓, GLUT4↑	1) Improved cardiac function 2) Activated mitophagy to reduce apoptosis 3) Promoted FA uptake	Cheng et al. (2020), Wang et al. (2020a), Zhou et al. (2020)
Qishen Granules (QSG)	1) Ligation of the LAD-induced HF in rats 2) Hypoxia-induced H9c2 cells	1) HIF-1α↑ 2) PPARα↑, RXRα↑, RXRβ↑, RXRγ↑, SUCLA2↑, CKMT2↑, UCP2↓, LDHA↓, PDK4↓ 4) CD36↑, CPT1A↑, ACADL↑, ACADM↑, ACAA2↑, SCP2↑	1) Improved cardiac function 2) Enhanced FA uptake and β-oxidation 3) Enhanced mitochondrial biogenesis	Gao et al. (2020), Sun et al. (2025)
Shenmai Injection (SMI)	1) Ligation of the LAD-induced post-MI HF in rats 2) H/R-induced H9c2 cells	1) PPARα/SIRT1/PGC1α signaling pathway↑ 2) BCL 2↑, BAX↓, IL-6↓, TNF-α↓, LDH↓, cTnI↓, ET-1↓	1) Improved cardiac function 2) Reduced inflammation and myocardial fibrosis 3) Enhanced the utilization efficiency of FFAs and improved lipid metabolism 4) Enhanced mitochondrial membrane potential 5) Inhibited mitochondrial autophagy	Yang et al. (2024)
Xinbao Pill (XBW)	1) Isoproterenol-induced HF in rats 2) Isoproterenol-induced NRCMs	1) AMPK/PPARα signaling pathway↑ 2) SGLT 1↓ 3) CD 36↑, CPT-1B↑, ACADM↑	1) Improved cardiac function 2) Ameliorated cardiac hypertrophy and dysregulation of fatty acid metabolism 3) Mitigated mitochondrial dysfunction	Pan et al. (2024)

### 5.3 Prospects of gene and cell therapies

Gene and cell therapies, as emerging treatment strategies, have demonstrated tremendous potential in restoring mitochondrial function and regulating fatty acid metabolism, offering new hope and directions for HF management.

#### 5.3.1 Gene therapy

Gene therapy involves introducing normal or therapeutic genes into a patient’s body to fix or replace defective genes, thereby achieving therapeutic effects. Recently, gene therapy has shown promising potential in treating mitochondrial dysfunction and fatty acid metabolic disturbances in HF (Linthout et al., 2024). A gene-editing platform based on tandem mitochondrial zinc finger nucleases (mtZFNs), delivered through adeno-associated virus (AAV), has been used to eliminate pathogenic mtDNA and reduce the heteroplasmic load selectively (Nash et al., 2025). This method effectively restored metabolic functions in both cardiac and skeletal muscle tissues in animal models without causing significant immune or inflammatory responses. Additionally, mitochondrial base editors, such as double-stranded DNA cytosine base editors (DdCBEs), have been created to introduce corrective edits that fix structural problems in mitochondrial tRNAs, thereby restoring mitochondrial function (Barrera-Paez et al., 2025). However, clinical application requires careful management of dosage and minimizing off-target effects. For more complex or unidentified mtDNA mutations, novel strategies using nanoparticle delivery of intact, healthy mtDNA have demonstrated success in cell models by achieving long-term expression of exogenous genes and sustained production of mitochondrial RNA and proteins (Wilson et al., 2025). These advancements offer a promising foundation for treating a wide range of mitochondrial disorders.

In the field of metabolic regulation, gene therapy can target key enzymes or signaling pathways to correct imbalances in FAO. For example, overexpressing an engineered bacterial lipoic acid ligase (lplA) can bypass defective acylation pathways, restore cellular respiration, and improve glucose intolerance, offering a potential treatment for acylation-related metabolic disorders (Bick et al., 2024). PPARα, a pivotal transcription factor that regulates fatty acid metabolism, has also emerged as a promising target (Barger and Kelly, 2000). Delivering PPARα plasmids specifically with a nanoparticle carrier (CMCP) repaired mitochondrial damage in pathological cardiac hypertrophy (Rana et al., 2019). Mechanistically, PPARα interacts with the C-terminal domain of p53, inhibiting its acetylation and disrupting the interaction between p53 and GSK3β. This stabilizes ΔΨm and reduces cardiomyocyte apoptosis. Additionally, overexpressing PPARα restored the expression of adipose triglyceride lipase (ATGL), a key enzyme in FAO, thereby enhancing myocardial energy metabolism and reversing pathological remodeling. This approach significantly improves gene delivery efficiency by utilizing the tissue-targeting properties of nanomaterials and has shown long-term cardioprotective effects in animal models. Notably, these mechanisms work synergistically with existing therapies for FAO disorders. For instance, AAV9-mediated delivery of the very longchain acylCoA dehydrogenase (VLCAD) gene was used to treat very long-chain acyl-CoA dehydrogenase deficiency, directly correcting the β-oxidation defect and maintaining stable cardiac function for up to 6 months in mice (Keeler et al., 2012). These findings indicate that gene interventions targeting different metabolic points can improve HF phenotypes by restoring mitochondrial function and metabolic homeostasis.

#### 5.3.2 Stem cell therapy

Stem cells possess the remarkable ability to self-renew and undergo multilineage differentiation, enabling them to develop into various cell types such as cardiomyocytes and vascular endothelial cells. This potential offers a promising therapeutic approach for HF. Among these cells, mesenchymal stem cells (MSCs) are among the most commonly used in regenerative therapies due to their immunomodulatory, anti-inflammatory, and tissue repair-promoting properties. Research on bone marrow-derived mesenchymal stromal cells (BMSCs) has shown that optimizing their metabolic profile, especially regarding energy utilization, can significantly improve their therapeutic impact (Ulmer and Eschenhagen, 2020; Jacques et al., 2024). For example, selectively enriching BMSCs subpopulations with glycolytic metabolic signatures has been demonstrated to increase their resistance to oxidative stress and improve post-transplant survival. This metabolic optimization has also been associated with increased left ventricular ejection fraction and decreased myocardial fibrosis in mouse models of doxorubicin-induced heart failure.

Importantly, mitochondrial bioenergetic maturation driven by fatty acid metabolism also plays a critical role in the success of stem cell therapies. Modulating fatty acid availability in the culture environment of human induced pluripotent stem cell-derived cardiomyocytes (hiPSC-CMs) has been demonstrated to promote mitochondrial remodeling, facilitate the assembly of respiratory chain complexes, and enhance ultrastructural maturation (Ramachandra et al., 2018). These changes help mimic the metabolic characteristics of late-stage cardiomyocytes, thus improving the functional integration and therapeutic potential of transplanted cells in cardiac repair.

#### 5.3.3 Mitochondrial transplantation

In recent years, mitochondrial transplantation has become a new therapeutic approach and has shown significant cardioprotective effects in different models of HF. Studies demonstrate that adding functional, intact mitochondria can effectively correct energy metabolism imbalances and oxidative stress caused by mitochondrial dysfunction. In a doxorubicin-induced model of dilated cardiomyopathy, transplanting mitochondria from various donor cell types—such as fibroblasts, skeletal muscle cells, and cardiomyocytes—substantially enhanced myocardial contractile function. However, the success of the therapy largely depends on the metabolic compatibility between donor mitochondria and recipient cardiomyocytes. Notably, in a cardiomyocyte model derived from induced pluripotent stem cells (iPSCs) of patients with genetically linked dilated cardiomyopathy, mitochondrial transplantation significantly improved ventricular contractility. Single-cell transcriptomic analysis showed that the enhanced myocardial function was mainly due to the restored mitochondrial respiratory activity, rather than widespread transcriptional alterations.

Animal studies have further confirmed the therapeutic potential of mitochondrial transplantation. In a right ventricular pressure overload model, direct intramyocardial injection of autologous skeletal muscle-derived mitochondria significantly reduced cardiomyocyte apoptosis (Weixler et al., 2021). It slowed the progression of right heart failure by maintaining the ΔΨm and ATP production capacity. Innovative research on acute toxic cardiomyopathy has shown that intravenous administration of healthy mitochondria can competitively bind toxic agents, thereby reducing oxidative damage (Shabani et al., 2024). This approach increased the survival rate of poisoned rats to 56.25% and greatly decreased myocardial injury. These protective effects may be connected to the “metabolic sponge” function of transplanted mitochondria and the regulation of mitophagy. It is worth noting that different transplantation routes (intracardiac injection/intravenous infusion) and mitochondrial sources (autologous/allogeneic) can influence therapeutic outcomes. Autologous mitochondria avoid immune rejection, while allogeneic mitochondria have demonstrated metabolic compensatory effects in specific experimental models.

### 5.4 Other therapeutic approaches

Exercise training is a nonpharmacological intervention that has beneficial effects on mitochondrial function and fatty acid metabolism. Studies have shown that long-term aerobic exercise, such as running or swimming, increases the mitochondrial content in cardiomyocytes, enhances the ΔΨm, and increases the activity of respiratory chain complexes, thus improving the energy supply to the myocardium (Shah et al., 2024; Hastings et al., 2023). Exercise training also modulates fatty acid metabolism by promoting fatty acid oxidation and reducing fatty acid uptake and storage. Moreover, exercise activates the AMPK signaling pathway, increases the activity of CPT1, and facilitates mitochondrial β-oxidation, thus accelerating fatty acid catabolism, reducing intracellular lipid accumulation, and mitigating lipotoxicity (Spaulding and Yan, 2022; Li et al., 2025). Exercise also improves insulin sensitivity and enhances insulin signaling, further contributing to the regulation of lipid metabolism.

Nutritional support is another crucial part of HF management. Appropriate nutritional strategies can improve mitochondrial function and support fatty acid metabolism. Omega-3 polyunsaturated fatty acids (ω-3 PUFAs), especially eicosapentaenoic acid (EPA) and docosahexaenoic acid (DHA), are known for their cardiovascular benefits (O’Connell et al., 2017). ω-3 PUFAs improve mitochondrial function by enhancing membrane fluidity and stability, which increases the activity of mitochondrial respiratory chain complexes and promotes ATP production. Additionally, ω-3 PUFAs help regulate lipid metabolism by blocking fatty acid synthesis and encouraging oxidation, lowering circulating triglycerides, reducing lipid buildup in cardiomyocytes, and alleviating lipotoxicity (Stanley et al., 2012). Supplementing with ω-3 PUFAs has been shown to enhance heart function in HF patients and decrease the risk of cardiovascular events. L-carnitine, a nutrient that aids in transporting fatty acids into mitochondria, assists β-oxidation and raises fatty acid oxidation rates, providing more energy to cardiomyocytes. Clinical research indicates that L-carnitine supplementation in HF patients raises myocardial ATP levels, improves LVEF, reduces (NT-proBNP levels, and improves overall clinical outcomes (Agrawal et al., 2022). Therefore, L-carnitine supplementation may enhance mitochondrial function and fatty acid metabolism in HF patients, supporting better cardiac performance and symptom relief.

### 5.5 Clinical evidence and translational implications

Clinical evidence for metabolic interventions in HF shows a layered landscape of maturity. Among these, SGLT2 inhibitors stand out as the most successful example, having advanced from mechanistic insights to multiple large-scale RCTs and guideline-recommended first-line therapy. Their benefits on hospitalization, mortality, and quality of life have been consistently demonstrated, and importantly, are independent of glycemic control. Exercise training and ω-3 PUFAs also have relatively strong clinical support, with several trials showing improvements in functional capacity and, in some cases, reductions in adverse outcomes. By contrast, TCM formulations and nutritional supplements such as coenzyme Q10 or L-carnitine remain in an earlier stage of clinical validation, with smaller RCTs suggesting potential benefits but lacking the robustness to alter international guidelines. Even further from clinical translation are gene-, cell-, and mitochondrial-based therapies, where evidence is still predominantly preclinical, though early signals are encouraging. A closer look at this landscape highlights the striking heterogeneity in the strength of evidence.

The rapid success of SGLT2 inhibitors reflects not only their strong mechanistic rationale but also broad applicability across HF phenotypes and the advantage of testing within large cardiovascular outcome trials originally designed for diabetes. In contrast, many studies on metabolic modulators, herbal formulations, or supplements have been constrained by small sample sizes, short durations, and reliance on surrogate endpoints such as NT-proBNP reduction or improvements in left ventricular ejection fraction. While these results are promising, they fall short of establishing definitive clinical efficacy. For emerging gene and mitochondrial approaches, technological barriers in delivery, long-term safety, and scalability remain major obstacles, leaving human data scarce.

The growing body of evidence linking mitochondrial dysfunction and impaired fatty acid metabolism to HF provides not only mechanistic insights but also translational opportunities for novel interventions. While pharmacological agents, natural products, and gene or cell therapies have demonstrated efficacy in preclinical studies, their clinical relevance depends on identifying actionable targets, suitable patient populations, and potential integration with existing therapies. PPARα–CPT1/ATGL axis to restore FAO flux; SIRT1/3 and AMPK/PGC-1α to promote mitochondrial biogenesis and quality control; NAD^+^ redox balance to improve electron transport efficiency; Pink1/Parkin–mediated mitophagy to remove dysfunctional mitochondria; and direct augmentation of electron transport (e.g., CoQ10, mitochondria-targeted peptides). These targets are pharmacologically tractable (small molecules, nutraceuticals, or biologics) and amenable to gene or nanoparticle approaches demonstrated in preclinical studies.

Given the heterogeneity of HF, metabolic interventions may be particularly effective in patients with defined phenotypes. Individuals with heart failure with preserved ejection fraction (HFpEF) often present with systemic metabolic disturbances, insulin resistance, and impaired FAO, suggesting a subgroup that may benefit from therapies enhancing mitochondrial efficiency. Conversely, heart failure with reduced ejection fraction (HFrEF) patients with ischemic or chemotherapy-induced cardiomyopathy may derive greater benefit from mitochondrial protectants or transplantation strategies. Patients carrying pathogenic mtDNA mutations or rare FAO enzyme deficiencies also represent a niche population for gene-based therapies. Stratifying patients by metabolic biomarkers, imaging signatures, or genetic profiles could help tailor interventions in a precision medicine framework.

Current guideline-directed therapies, such as SGLT2 inhibitors, have already established the feasibility of metabolic modulation in HF, while exercise, ω-3 PUFA, and L-carnitine supplementation provide additional support through effects on mitochondrial and FAO pathways. TCM formulations, including QLQX and SMI, have also demonstrated clinical benefits, suggesting their value as adjunctive options. Given the multifactorial nature of HF, single-target interventions are unlikely to suffice, and multimodal strategies combining pharmacological agents, exercise, dietary approaches, and nutraceuticals may offer synergistic effects. For example, SGLT2 inhibitors enhance substrate utilization while exercise promotes mitochondrial biogenesis, together reinforcing myocardial energy homeostasis. Although still exploratory, gene and cell therapies fit into this integrative paradigm by directly repairing mitochondrial defects.

## 6 Discussion

HF remains a global health burden, with mitochondrial dysfunction and fatty acid metabolic disturbances increasingly recognized as central mechanisms in its progression. Preclinical studies and clinical trials demonstrate that targeting metabolic pathways can improve cardiac remodeling, functional capacity, and long-term outcomes. These findings highlight metabolic modulation not only as a mechanistic rationale but also as a clinically feasible therapeutic strategy.

Notably, mitochondrial dysfunction and disordered fatty acid metabolism act in a bidirectional manner. On one side, impaired oxidative phosphorylation reduces FAO, leading to ATP deficiency and contractile failure. On the other, excessive fatty acid uptake and incomplete β-oxidation generate toxic intermediates and oxidative stress, destabilizing mitochondrial membrane potential and further impairing mitochondrial function. This vicious cycle accelerates maladaptive remodeling, underscoring the need for therapies that jointly preserve mitochondrial integrity and restore FAO homeostasis.

Despite promising evidence, important gaps remain. Direct clinical studies on mitochondrial function and FAO in HF are limited, with most interventional trials relying on surrogate endpoints rather than hard outcomes. Patient heterogeneity further complicates translation: HFpEF is frequently associated with systemic metabolic dysfunction, whereas HFrEF often reflects ischemic or toxic injury. Comorbidities such as diabetes and obesity add further complexity, yet few studies incorporate metabolic stratification. Additionally, many existing trials are restricted by small sample sizes, short duration, or lack of long-term follow-up.

Future research should address these limitations along three directions. First, precision phenotyping that integrates metabolomics, imaging, and genetic profiling is needed to define metabolic subgroups and guide tailored interventions. Second, multimodal strategies should replace single-target approaches. Combining pharmacological agents, structured exercise, dietary modulation, and potentially nutraceuticals or traditional Chinese medicine may yield synergistic benefits; for instance, SGLT2 inhibitors improve substrate utilization while exercise promotes mitochondrial biogenesis. Third, gene- and cell-based therapies, though still exploratory, hold promise for repairing mitochondrial defects or enhancing bioenergetic capacity, provided challenges in delivery, safety, and durability can be overcome. Finally, future trials must prioritize clinically meaningful endpoints—including mortality, hospitalization, and quality of life—while adopting pragmatic designs and long-term follow-up.

In summary, mitochondrial dysfunction and FAO disturbances form a self-reinforcing cycle that drives HF progression. Bridging mechanistic discoveries with patient-centered outcomes through precision phenotyping, integrative therapies, and innovative trial design may ultimately establish metabolic modulation as a cornerstone in HF management.
